# Vitamin D modulates gene expression in four major muscle tissues in Atlantic salmon

**DOI:** 10.1038/s41598-025-22850-1

**Published:** 2025-11-06

**Authors:** Courtney E. Gorman, Fintan Egan, Francisco Javier Alarcón-López, Jette Jakobsen, Philip McGinnity, C. Darrin Hulsey

**Affiliations:** 1 School of Biology and Environmental Science, University College Dublin, Belfield, Dublin, Ireland; 2 Marine Institute, Newport, Ireland; 3 Department of Biology and Geology, University of Almería, 04120 Almería, Spain; 4 National Food Institute, Technical University of Denmark, 2800 Lyngby, Denmark; 5 School of Biological, Earth and Environmental Sciences, University College Cork, Cork, Ireland

**Keywords:** Biofortification, Aquaculture, Modularity, Salmonid, Nutrigenomics, Gene expression, Ichthyology

## Abstract

Vitamin D is essential to muscle health but could affect different muscle tissues in distinct ways. In humans, vitamin D is primarily synthesized in the skin via sun exposure but can also be obtained through ingesting foods that are fortified (e.g., milk) or naturally high in vitamin D (e.g., salmon). Like humans, salmon vitamin D content can vary considerably and can be altered through dietary consumption of vitamin D. We experimentally determined how vitamin D manipulation alters gene expression in four salmon muscle tissues: skeletal, craniofacial, smooth, and cardiac. Vitamin D induced changes in expression were exceptionally profound in the heart, where there were also several gene duplicates expressed unidirectionally. Five genes were differentially expressed in multiple muscles and many genes impacted by our vitamin D treatments in salmon show extensive evolutionary conservation as they are influenced by vitamin D titers in mammals, including humans. Salmon could provide a powerful model for understanding how variation in vitamin D impacts all vertebrates.

**Supplementary Information:**

The online version contains supplementary material available at 10.1038/s41598-025-22850-1.

## Introduction

Vitamin D is crucial to health. While vitamin D is well known for its role in skeletal function, vitamin D deficiency is also associated with a wide range of human health problems including cardiovascular disease, cancer, COVID-19, immune dysfunction, tooth decay, and digestive disorders^1–3^. It is increasingly clear that vitamin D also plays an important role in muscle^4,5^. However, most studies to date have investigated the aggregate effects of vitamin D on skeletal muscle mass and strength^6–8^. Much less is known about how vitamin D may up- or downregulate specific genes and proteins important for muscle function^9^ or how different muscle tissue types (e.g., cardiac and smooth muscle) might be impacted by increases in vitamin D. Because invasive, destructive sampling limits the feasibility of examining multiple muscle tissues in human studies, there is a clear need for animal models that enable tissue-specific investigations of vitamin D. Atlantic salmon (*Salmo salar*) provide a particularly useful model, as they require vitamin D for normal physiological function, which they are thought to obtain exclusively from dietary sources. This makes these fish especially responsive to manipulations of vitamin D intake. In this study, we experimentally manipulated vitamin D levels in Atlantic salmon to determine how supplementation influences gene expression in four distinct muscle tissues.

Vitamin D rich salmon have high nutritional value to humans. Although humans primarily synthesize vitamin D in the skin through sun exposure^10^, dietary sources such as fatty fish like salmon also contribute to vitamin D status^11^. Given that altitude, weather, and sun-avoidance can all limit human vitamin D synthesis^10^, dietary sources have long been important to human health. In recent years, biofortification has emerged as an attractive strategy to combat vitamin D deficiency^10,12^, especially in regions with limited sunlight. This approach involves increasing the vitamin D content of animal-derived foods, such as eggs or fish, by adding vitamin D to animal diets. As a fatty fish, salmon are often naturally high in vitamin D and are one of the few animals that can vary substantially in the vitamin D_3_ content of their muscles^13^. Salmon vitamin D content has been shown to vary based on geography and whether fish were farmed or wild-caught^13^. This variability is likely attributable to diet differences as salmon are not thought to synthesize vitamin D but rather accumulate it from trophic resources^14,15^. It is therefore biologically tractable and relatively similar to how salmon obtain vitamin D naturally to biofortify farmed salmon by manipulating vitamin D in their diet^13,16,17^. Although data is limited, no detrimental effects on salmon skeletal or hematological systems have been reported in response to increasing vitamin D levels^16,17^. Salmon are particularly well-suited for biofortification.

However, efforts to biofortify salmon and understand its consequences for gene expression should clarify how vitamin D in feeds subsequently accumulates in salmon. Vitamin D in nature exists in two main forms, vitamin D_2_ (ergocalciferol), mainly found in plants, and vitamin D_3_ (cholecalciferol), predominately found in animals. Vitamin D_3_ is the main form found in vertebrates, from fish to humans, and can be obtained directly through dietary sources^15^. Like terrestrial animals, salmon convert dietary vitamin D_3_ to 25-hydroxyvitamin D_3_ (25(OH)D_3_) and then into 1,25-dihydroxyvitamin D_3_ (1,25-OH-D_3_, calcitriol)^15,18^. In contrast to mammals, where the second hydroxylation occurs primarily in the kidney, salmonids carry out both steps primarily in the liver^15^. However, this second step may also occur in other tissues like the kidney, intestines, gills, and even muscle^15,18–20^. Fish often exhibit higher plasma concentration levels of vitamin D_3_ metabolites than mammals^21,22^, and are capable of storing large quantities of vitamin D_3_ in tissues such as the liver and fat without catabolizing it^15^. In contrast, mammals excrete excess D_3_ metabolites^23^. While their ability to store this nutrient makes salmon an excellent source of dietary vitamin D for humans, it remains unclear how the vitamin D that accumulates in salmon tissues interacts with muscle physiology and transcriptional regulation.

Vitamin D supplementation could impact muscles in many ways. For example, vitamin D supplementation improves skeletal muscle protein synthesis in vitamin D deficient rodents by changing expression of genes needed for maintenance and growth^24^. In human muscle cells, vitamin D also strongly impacts mitochondrial function and dynamics that modulate ATP necessary for muscle strength^25^. The vitamin D associated endocrine system of salmon could function and influence muscle in very similar ways to how it operates in mammals^15^. As many genes found in both salmon and human genomes likely have conserved functions, how salmon gene expression responds to changes in vitamin D could illuminate the way that vitamin D affects vertebrates in general. Because vitamin D concentration varies extensively in salmon muscle and manipulating it is relatively straightforward^13^, salmon vitamin D biofortification provides an ideal opportunity to understand how vitamin D affects gene expression in several muscle tissues that are conserved elements of the vertebrate body plan.

In both salmon and humans, there are at least three main types of muscle tissue, skeletal, smooth, and cardiac. Although contraction driven movement characterizes all muscles, muscle types differ in their cellular components, physiology, and specific functions^26^. Skeletal muscles like the filets obtained from fish axial musculature help maintain posture, power voluntary movement, and contribute to energy metabolism^27^. All skeletal muscle also consist mostly of individual actin and myosin fibers bundled together into a muscle spindle, but craniofacial muscles could have distinct responses to vitamin D as compared to other skeletal muscle^28^. For instance, facial muscles such as the adductor mandibulae (AM) that close vertebrate jaws could be quite transcriptionally differentiated from the axial skeletal muscles constituting most fish filets^28,29^. Smooth muscles are likely to be even more different. These types of muscles are also composed of actin and myosin fibers but are arranged in sheets and not striated like skeletal muscles. Smooth muscles that line many organs, such as the stomach, use contractile forces to propel and regulate the flow of food and other materials in the body^30^. Cardiac muscle, composed of individual cardiomyocytes, is striated like skeletal muscle and also contains cytoskeletal and contractile elements that are connected through intercalated discs^31^. However, unlike the skeletal and smooth muscle powering other organs, the involuntary muscle that encloses the heart’s chambers is constantly contracting and is the primary pump ensuring the blood maintains and oxygenates every cell in the body^31^. Since many vitamin D target genes could be largely tissue specific^32^, examining multiple muscles should allow us to understand how vitamin D impacts muscle gene expression both comparatively and in general.

Vitamin D augmented gene expression in Atlantic salmon muscles could be heavily influenced by the relatively recent whole genome duplication event that occurred in the common ancestor of all salmonids ~ 90 million years ago^33^. This type of whole genome duplication characterizing salmon and their close relatives might often fuel diversification and adaptive elaboration of important gene networks like those related to vitamin D^34,35^. However, following whole genome duplication, salmon developmental genomics could have been heavily influenced by rediploidization, the process of evolving back to a more diploid-like gene content^36^. Because of the redundancy in how and where duplicated genes were expressed, many duplicate genes that originated from the salmonid-specific genome duplication were subsequently lost^37^. In contrast, duplicate genes retained in salmon genomes and regulated by essential nutrients like vitamin D might be predicted to have frequently extensively diverged in their expression^38,39^. Understanding how duplicated genes respond to vitamin D supplementation could shed light on duplicate gene retention and provide additional pseudo-independent glimpses into conserved genes that respond to vitamin D in salmon muscles.

**Fig. 1:**
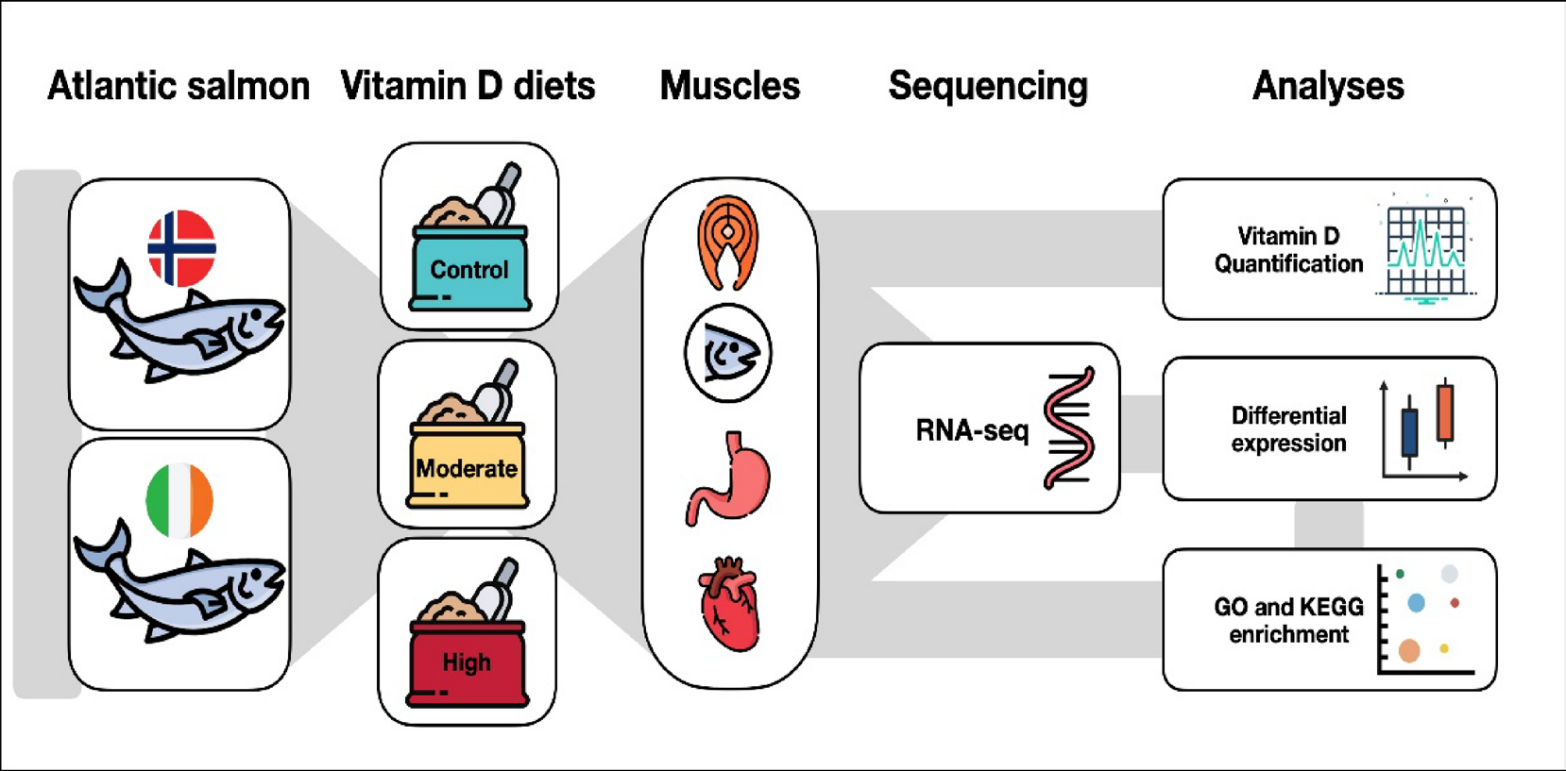
Overview of experimental workflow. Two strains of salmon were raised in experimental ponds and the level of vitamin D (control, moderate, and high) in their diets was manipulated for six months. Four major muscle tissues, filet (axial skeletal muscle), adductor mandibulae (craniofacial skeletal muscle), stomach (smooth muscle), and heart (cardiac muscle), were sampled to determine how vitamin D alters gene expression in salmon muscles. The filet was also used to determine the level of vitamin D accumulation in the muscle at the end of the experimental diets. The heart was further used for GO and KEGG enrichment analyses.

Here we aim to clarify how vitamin D affects vertebrate muscle in general and what impacts vitamin D supplementation has on muscle gene expression in the commonly consumed Atlantic salmon. We fed two strains of salmon diets with three levels of vitamin D: control (0 μg/kg), moderate (100 μg/kg), and high (1000 μg/kg) and measured the effects on salmon body condition (i.e., Fulton’s condition factor, or the ratio of body weight and body length cubed). The range of vitamin D doses were chosen to represent the extent of vitamin D supplementation that encompasses the natural variation found in Atlantic salmon as well as country-specific guidelines for vitamin D biofortification^40^. Then, we examined: (1) whether vitamin D supplementation influenced gene expression in the filet (axial skeletal muscle), adductor mandibulae (craniofacial skeletal muscle), stomach (smooth muscle), and heart (cardiac muscle), (2) if there are common ways vitamin D influenced gene expression across the muscle tissues and (3) whether any vitamin D induced changes in gene expression are conserved across vertebrates (see Fig. 1 for an overview of the experimental workflow).

## Results

### Vitamin D content

**Fig. 2:**
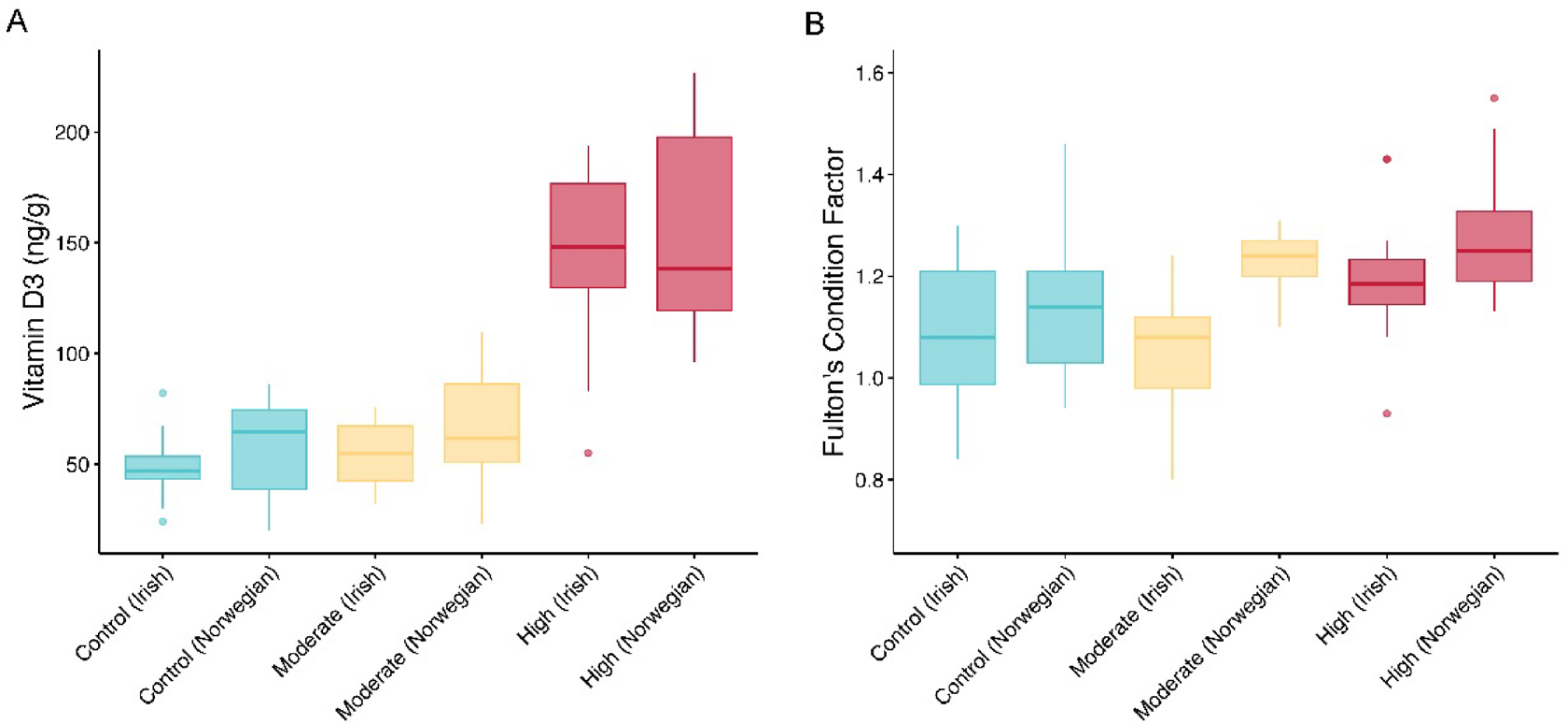
Boxplots of vitamin D quantification (**A**) and Fulton’s condition score (**B**). Vitamin D augmentation was similar in both Irish and Norwegian salmon strains and associated with better salmon condition at the end of the 6 months experimental treatments.

Vitamin D content varied extensively in our experimental feeds and fish. Among the experimental feeds, vitamin D_3_ was present in the following concentrations: control (729 ng/g), moderate (866 ng/g), and high (1779 ng/g). In the salmon filets, we quantified both vitamin D_3_ and its metabolite, 25-hydroxyvitamin D_3_ (25(OH)D_3_). All samples contained less than 1 ng/g of 25(OH)D_3_, with the exception of a single sample from the high vitamin D group, which had 1.4 ng/g. In contrast, vitamin D_3_ levels were substantially higher and showed clear treatment effects. The vitamin D_3_ concentrations ranged as follows: 24–86 ng/g (mean = 53.15, SD = 19.65) in the control, 32–110 ng/g (mean = 60.15, SD = 22.32) in the moderate, and 55–227 ng/g (mean = 148.1, SD = 45.03) in the high filets. Vitamin D_3_ content was significantly different among the salmon vitamin D treatment groups (ANOVA, *F*_2,56_ = 58.25, *p* ≤ 0.001, Fig. 2A). Tukey tests indicated vitamin D_3_ content was greater in the high vitamin D treatment compared to the moderate (adjusted *p* ≤ 0.001) and control (adjusted *p* ≤ 0.001) groups. The Irish and Norwegian strains did not significantly differ in vitamin D accumulation in the filet.

### Body condition

The mean Fulton’s body condition score was significantly different among strains (ANOVA, *F*_1,120_ = 19.42, *p* ≤ 0.001) and the vitamin D treatment groups (ANOVA, *F*_2,120_ = 12.54, *p* ≤ 0.001, Fig. 2B). Tukey tests indicated that the Norwegian strain had a significantly higher condition score than the Irish strain (adjusted *p* ≤ 0.001) that was driven by a slight difference in condition between the strains in the moderate treatment group (Fig. 2B). Post-hoc tests revealed that body condition was significantly higher in the high vitamin D treatment compared to the moderate (adjusted *p* ≤ 0.001) and control (adjusted *p* ≤ 0.001) groups (Fig. 2B).

### RNAseq libraries

The post-cleaned RNAseq libraries ranged from 10.86G to 16.86G (Supporting Information 1) with a mean of 12.56G and standard deviation of 1.19. Mapping rates ranged from 79.8 to 89.9%, with an average mapping rate of 85.7% (Supporting Information 2).

### Vitamin D influenced gene expression in all four muscle types

The greatest differences were found between the highest vitamin D levels and the control. Therefore, we comment on the differences in expression between the control and intermediate levels of vitamin D but focus on differential expression between the two extreme levels of vitamin D. Because we did not find differences in the accumulation of vitamin D between the two strains in the filet, we did not further examine the inter-strain differences in gene expression.

**Fig. 3:**
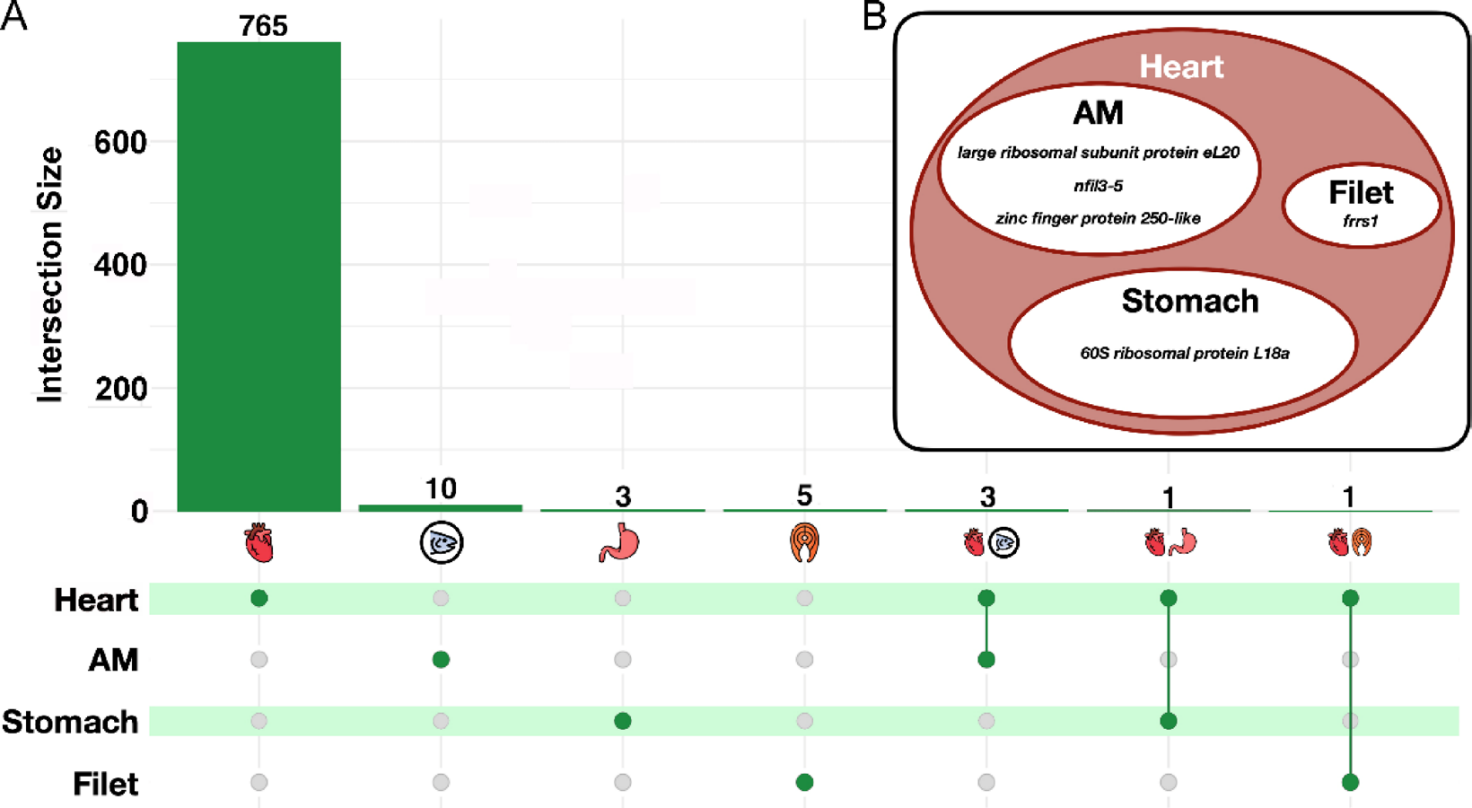
(**A**) Upset plot showing the numbers of differentially expressed genes in each muscle tissue between the control and high vitamin D treatments as well as the genes that were differentially expressed in more than one muscle tissue. The vertical bars (dark green) represent the number of differentially expressed genes for each muscle tissue combination. The horizontal bars with dots show which muscle tissue combination is represented by the vertical bars. The majority of differentially expressed genes were in the heart. (**B**) Euler diagram detailing the genes that were differentially expressed in the heart as well as another muscle tissue.

In the filet, there were no genes that were significantly differentially expressed (adjusted *p* ≤ 0.05) between the moderate group and the control group (Supporting Information 3). However, six genes were differentially expressed between the high and control groups (Table 1, Fig. 3). All six genes were significantly upregulated, with log_2_ fold changes (log_2_FC) greater than 1, indicating at least a two-fold increase in expression in the high vitamin D group compared to the control. Notably, EX001874 and EX004487 had log_2_FC greater than 4, corresponding to approximately 16-fold higher expression in the vitamin D supplemented filets.

**Table 1:** Summary of strongly differentially expressed genes (adjusted *p* ≤ 0.05 and absolute log_2_FC > 1) between the high and control groups across all four muscle tissues – filet, adductor mandibulae (AM), stomach, and heart.

Upregulated genes				Downregulated genes			
Muscle	Gene ID	Fold change (log_2_)	Gene name	Muscle	Gene ID	Fold change (log_2_)	Gene name
Filet	EX004487	4.4		AM	EX090080	− 3.8	
Filet	EX001874	4.2		AM	EX112871	− 3.1	*zinc finger protein 250-like*
Filet	EX091598	3.8	*upk1a*	AM	EX118289	− 1.6	
Filet	EX104459	3.7		AM	EX044965	− 1.5	*psat1*
Filet	EX120674	2.4		Stomach	EX089520	− 5.1	*protein BUD31 homolog*
Filet	EX059264	2.4	*frrs1*	Heart	EX073978	− 1.8	*slc24a4b*
AM	EX106621	2.9		Heart	EX073280	− 1.6	*klf15*
AM	EX116233	1.7		Heart	EX007137	− 1.6	*cox4i2*
AM	EX099524	1.4	*nfil3-5*	Heart	EX064884	− 1.5	*chordc1a*
AM	EX005415	1.2		Heart	EX000959	− 1.5	*gys1*
Stomach	EX089520	5.1		Heart	EX069706	− 1.4	*cp1a1*
Heart	EX059264	8.1	*frrs1*	Heart	EX042664	− 1.4	*gys1*
Heart	EX073472	1.9	*si:ch73-206d17.1*	Heart	EX004801	− 1.3	*lrat*
Heart	EX002465	1.5	*emp1*	Heart	EX007690	− 1.3	*acta1*
Heart	EX065996	1.5	*zgc:73226*	Heart	EX066339	− 1.3	*slc2a1b*
Heart	EX115603	1.5	*tnnI1*				
Heart	EX054208	1.3	*ate1*				
Heart	EX088475	1.3	*gas2a*				
Heart	EX077051	1.3	*tulp1b*				
Heart	EX064919	1.2	*gamt*				
Heart	EX057658	1.2	*zgc:162297*				

Due to the large number of genes differentially expressed in the heart, only the ten most upregulated and downregulated genes that could be annotated are shown for the heart. The full list of differentially expressed genes can be found in Supporting Information 4. A shortened form of the Ensembl ID is given for each gene where “EX” has replaced “ENSSSAG00000”.

In the adductor mandibulae, there was only one gene (EX094662) upregulated when the control was compared to the moderate group (Supporting Information 3). However, 13 genes were differentially expressed (adjusted *p* ≤ 0.05) between the high vitamin D group and the control group (Fig. 3). Four genes were upregulated and nine were significantly downregulated in the vitamin D supplemented group. All four upregulated genes and four of the nine downregulated genes had absolute log_2_FC greater than one (Table 1). The most upregulated gene was EX106621 (fold change (log_2_) = 2.9) and the most downregulated gene was EX090080 (fold change (log_2_) = − 3.8). Annotation for both genes could not be determined via our BLAST search.

Only one gene (EX105051) was differentially expressed in the stomach muscle between the moderate and control groups (Supporting Information 3). Four genes were differentially expressed between the high vitamin D group and the control group in the stomach (Table 1, Fig. 3). Of these, only one had an absolute log_2_FC greater than 1 (Table 1). The gene EX089520, identified as a protein BUD31 homolog via NCBI, had a log_2_FC of 5.1, indicating that its expression was approximately 34-fold higher in the vitamin D group compared to the control.

There were eight genes that were differentially expressed between the moderate and control groups in the heart, four of which had log_2_FC greater than one (Supporting Information 3). Three of the genes found to be differentially expressed between the high and control groups were also significantly differentially expressed between the moderate and control groups (Supporting Information 3). The genes *gas2a* and EX122198 were upregulated and *mtfr1l* was downregulated in response to both moderate and high vitamin D treatments in the heart (Supporting Information 3 and 4).

In contrast to the other muscles, 770 genes were differentially expressed between the high vitamin D group and the control in the heart (Fig. 3). Of these, 59 had an absolute log_2_FC greater than one. There were 347 genes that were upregulated and 423 that were downregulated (Table 1, Supporting Information 4). The most upregulated gene was *frrs1* (putative ferric-chelate reductase 1), with a log_2_FC of 8.1, indicating a 274-fold increase in the high vitamin D group compared to the control. Notably, this gene was also strongly upregulated (log_2_FC = 2.4) in response to high vitamin D supplementation in the filet. The most downregulated gene, identified via NCBI as tetratricopeptide repeat protein 30A, had a log_2_FC of − 2.0 (Table 1, Supporting Information 4).

### GO and KEGG enrichment analyses

**Fig. 4:**
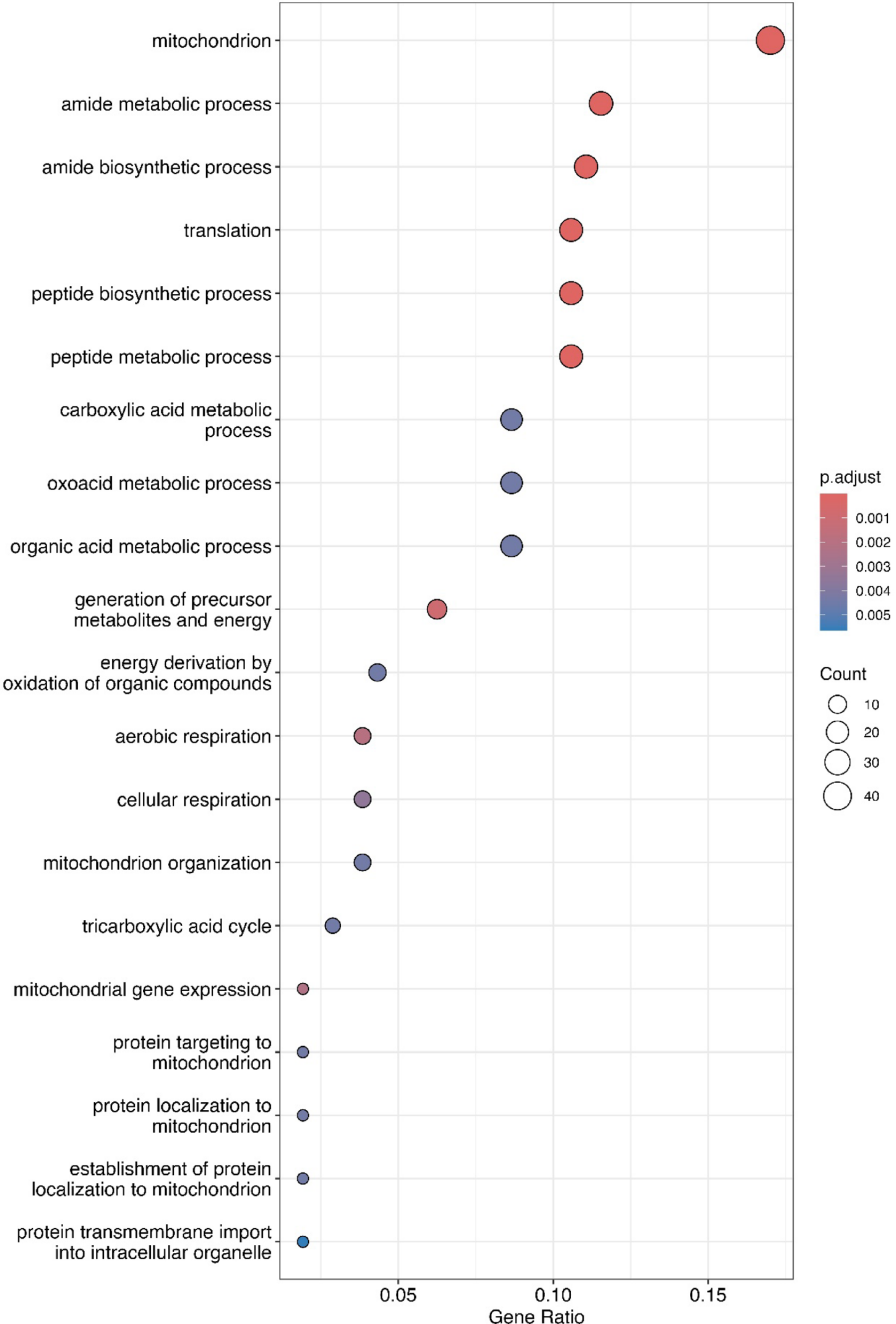
Dot plot showing the top 20 GO (gene ontology) terms from the GO enrichment analysis of the several hundred differentially expressed heart genes in response to higher vitamin D. The circle sizes reflect the gene counts assigned to particular GO categories, and circle colors represent the adjusted *p*-value significance The gene ratio along the x-axis represents the number of differentially expressed genes adjusted by the number of genes associated with that GO term in the salmon genome.

Given the large number of differentially expressed genes in the heart between the high and control vitamin D treatments, we performed gene ontology (GO) analyses to further characterize these effects of vitamin D on differential gene expression. We found that 37 GO categories were significantly enriched: 19 for biological processes (BP), 16 for cellular components (CC), and two for molecular functions (MF). The GO category with the highest gene count was ‘mitochondrion’ (Fig. 4). Further, by mapping the differentially expressed genes to the KEGG database of metabolic pathways, we identified seven pathways that were significantly enriched (adjusted *p* ≤ 0.05) (Supporting Information 5). The enriched pathways that had the highest gene counts were the ‘ribosome’ and ‘carbon metabolism’ pathways (Supporting Information 5).

### Pleiotropic differential expression across muscles

**Fig. 5:**
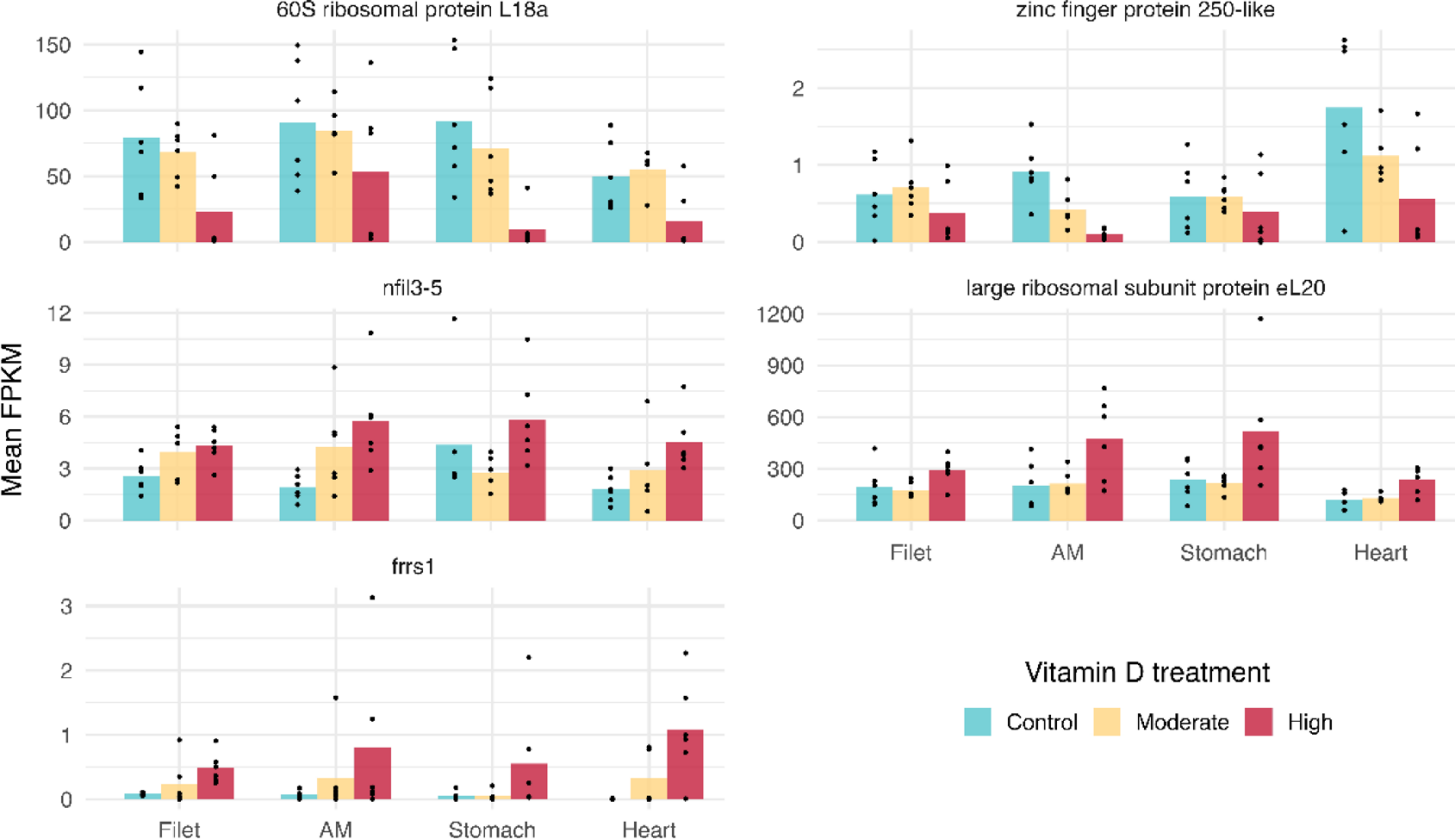
Mean Fragments Per Kilobase of transcript per Million mapped reads (FPKM) for the six genes that were differentially expressed in more than one muscle tissue (see Fig. 3 for details). The genes EX009988 (60S ribosomal protein L18a) and EX112871 (zinc finger protein 250-like) were expressed more highly in the control treatment (blue) and least expressed in the high vitamin D treatment (red). In contrast, the genes *nfil3-5* and EX005415 (large ribosomal subunit protein eL20) were upregulated in response to the high vitamin D treatment and expressed less in the control treatment. The gene *frrs1* had little to no expression in the control and moderate vitamin D treatment (yellow) and higher expression in response to the high vitamin D treatment. In general, despite differential expression only being found to be statistically significant in two muscles, expression of these six genes followed similar patterns across the vitamin D treatments in all four muscle tissues.

Five genes were differentially expressed (adjusted *p* ≤ 0.05) in more than one muscle tissue (Fig. 3). These pleiotropically differentially expressed genes showed clear patterns as they were either least expressed in the control treatments and most expressed in the high vitamin D treatments, or vice versa (Fig. 5). One of the shared genes, *frrs1,* had little to no expression in the control and moderate vitamin D treatments but had much more substantial expression in the high vitamin D treatments (Fig. 5).

### Duplicate genes

We found 41 gene duplicates and one gene triplicate (i.e., 85 unique ensemble IDs which corresponded to 42 genes) to be differentially expressed (Supporting Information 6). Interestingly, gene duplicates were always differentially expressed in the same direction. Both copies were either up- or downregulated, and it was never the case that one copy was upregulated while the other was downregulated.

### Gene homology of the genes with mammals

Our NCBI gene and BLAST searches enabled us to annotate an additional 216 out of 291 unnamed genes from Ensembl (Supporting Information 3). The literature search identified 12 genes that were both differentially expressed in our dataset and have recognized gene expression differences in muscle associated with vitamin D levels in humans and other mammals (Table 2).

**Table 2:** List of genes that returned studies after querying PubMed with the search terms: “[gene name]” AND expression AND muscle AND ‘vitamin D’”.

Gene	Gene description	Organism
*Sod1*	Superoxide dismutase 1	Mice^73^ Rats^74–76^
*Opa1 / OPA1*	OPA1 mitochondrial dynamin like GTPase	Mice^77,78^ Rats^79^ Human skeletal muscle cells^25^
*Fbxo32*	F-box protein 32	Rats^24^ Mice^80,81^
*Foxo3 / FOXO3*	Forkhead box O3	Mice^82^ Human skeletal muscle cells^83^
*Ppara*	Peroxisome proliferator-activated receptor alpha	Rats^84^
*Cs*	Citrate synthase	Mice^77,85^ Rats^24^
*Bmi1*	BMI1 Proto-Oncogene	Mice^86^
*Bnip3*	BCL2 interacting protein 3	Mice^81^
*EPAS1*	Endothelial PAS domain protein 1	Human skeletal muscle cells^25^
*FIS1*	Fission, mitochondrial 1	Human skeletal muscle cell^25^
*Hmgb1*	High mobility group box 1	Mice^87^
*YY1*	YY1 Transcription Factor	Human mammary fibroblast^88^

The focal organism as well as the of the studies are also given.

## Discussion

Vitamin D supplementation favorably increased body condition and influenced gene expression in all four muscle tissues. Gene expression in the heart was particularly impacted by the high vitamin D treatment, with 770 significantly differentially expressed genes compared to four in the stomach, six in the filet, and 13 in the adductor mandibulae. Our vitamin D quantification revealed that while the moderate vitamin D feed contained more vitamin D than the control, the vitamin D levels were relatively similar between the control and moderate treatments. This likely explained the overall muted gene expression responses to the moderate treatment. We also found five genes that were differentially expressed in more than one muscle tissue in response to the high vitamin D treatment. Several gene duplicates were similarly expressed across multiple muscle tissues, and these were always found to be expressed in the same direction. Finally, we identified several genes impacted by vitamin D in salmon that are known to be impacted by vitamin D in mammals. These findings help clarify the conserved impacts of vitamin D supplementation on gene expression in vertebrate muscle generally and underscore the importance of investigating the consequences of vitamin D augmentation on multiple tissues simultaneously.

Our vitamin D biofortification of the salmon via their diet led to an increase in vitamin D in the muscle making up their filet (Fig. 2A). Despite several documented biological differences between the Irish and Norwegian strains^41^, their accumulation of vitamin D in the filet did not differ. Also, while no detrimental impacts of vitamin D supplementation have been previously found in salmon skeletal or hematological systems^16,17^, vitamin D bioaccumulation could have unintended negative effects. However, our vitamin D supplementation led to a higher condition factor in the salmon consistent with a positive health boost to the salmon (Fig. 2B). Vitamin D is known to facilitate muscle hypertrophy and regeneration as well as prevent muscle atrophy in mammals^5,42^. Although whether the increased condition factor of the fish was due to changes in body fat, bone mass, or an increase in the mass of a range of muscles should be examined more extensively. Regardless, it would be a win–win for the vitamin D biofortification of salmon if this supplementation not only provided a more vitamin D dense food for humans but also positively enhanced body condition of important organ systems in salmon like their skeletal, smooth, and cardiac musculature.

Vitamin D is now recognized to play multiple roles in muscle development and maintenance^9,43,44^. However, a given ingested amount of vitamin D could generally influence different muscle tissues in highly divergent ways. Notably, disparate effects were recovered here for the effects of vitamin D on different salmon muscle tissues. The much greater number of differentially expressed genes in the heart compared to the other muscle types demonstrate the importance of examining the influence of vitamin D titers on multiple tissues simultaneously (Fig. 3). Unfortunately, based on our experimental design it is difficult to know whether vitamin D might preferentially bioaccumulate in cardiac muscle or if our vitamin D treatments reset the gene expression landscape of cardiac tissue to a greater degree than the other muscles. However, our results do suggest that long term vitamin D supplementation can have substantial effects on gene expression. In the future, increased efforts should be made to examine how particular vitamin D titers and the timeframe over which they are manipulated influence disparate muscle tissues. Distinct muscles may respond quite differently when vitamin D augmentation is used for enhancing biofortification, increasing animal well-being, or directly as a diet supplement for improving human health.

Despite clearly influencing gene expression in all four muscle tissues examined (Fig. 3), vitamin D augmentation had highly divergent effects across tissues. Nevertheless, there was evidence of pleiotropic differential expression, with some genes differentially expressed in more than one muscle tissue type (Fig. 3). For example, the genes EX112871, identified as zinc finger protein 250-like via NCBI, and EX009988 (60S ribosomal protein L18a) were downregulated not only in the heart, but in the adductor mandibulae and stomach, respectively. The genes EX005415 (large ribosomal subunit protein eL20) and *nfil3-5* were upregulated in the adductor mandibulae and heart, while *frrs1* was strongly upregulated in the heart and the filet. This gene, a putative ferric-chelate reductase 1, likely plays a role in the uptake of iron, which is an essential nutrient for almost all organisms^45^. Studies in humans have demonstrated links between iron and vitamin D metabolism, however the underlying mechanisms remain unclear^46^. These findings highlight the broad physiological role of vitamin D and suggest it may mediate pleiotropic effects across multiple tissues.

The exceptional divergence in gene expression found in the salmon heart was surprising. However, the metabolic intensity of the heart coupled with the importance of vitamin D’s role in modulating calcium might underlie the outsized influence of our vitamin D treatments on heart gene expression^47,48^. Interestingly, our GO and KEGG enrichment analyses in the heart both indicated a disproportionate effect of the vitamin D supplementation on protein synthesis and mitochondrial associated functions (Fig. 4). Vitamin D has been documented to greatly influence mitochondrial function^25,49,50^. As vitamin D is known to play a role in reducing oxidative stress and mitochondria are particularly susceptible to oxidative stress^51–53^, the downregulation of mitochondria-related genes we observed could be related to greater vitamin D availability ameliorating this stress in the salmon hearts. Because of the evolutionary conservation and importance of the heart to vertebrate evolution, salmon could provide a powerful model for understanding how vitamin D influences long-term heart functional health.

Although genome duplication has occurred more recently in salmonid fishes, the genomes of all vertebrates have been structured by several rounds of genome duplication^33–35^. The resultant doubling of the genes in the genome likely have had profound effects on vertebrate gene expression especially when adapting to novel challenges such as new nutritional regimes^54^. Following the type of genome duplication found in salmonids, many gene duplicates might be readily lost or co-opted for novel roles due to their functional redundancy in gene networks^38,39^. However, we found exceedingly consistent similarity in the direction of differential expression in salmon gene duplicates in response to our salmon vitamin D supplementation (Supporting Information 6). Gene duplicates that are retained might often be expected to experience subfunctionalization or neofunctionalization of their ancestral role in the gene expression landscape^55^. This type of divergence has been found in many organisms, including salmon, following genome duplication^36,37^. In contrast, we found substantial evidence for conserved responses of duplicate genes to an increase in vitamin D. Every set of the 42 duplicated genes showing differential expression in response to an increase in vitamin D increased or decreased expression in the same direction (Supporting Information 6). Gene duplicates could often diverge in what tissues they are found or when during ontogeny they are expressed^38^. However, when gene duplicates are retained for an important endocrine pathway like that associated with vitamin D, it may be that these retained duplicates rarely diverge in how they plastically respond to changes in essential nutrients.

The conservation of vitamin D’s effects on various aspects of muscle gene expression highlights the similar nature of vitamin D’s impacts on muscle function across vertebrates. Understanding the conserved biological consequences of vitamin D supplementation could provide more confidence and insight into the effects of vitamin D not only on human health but also on biofortification of other animals including salmon. As vitamin D fortification of human protein sources like salmon is becoming more common, it is critical to evaluate whether vitamin D supplementation will have predictable and potentially even positive consequences on the well-being of these animals. For instance, additional studies in salmon could provide a powerful model for how vitamin D influences organs such as the heart with respect to age, geographic origin of salmon populations, or whether fish are captive bred or wild caught. The insights gained and synergistic consequences of vitamin D supplementation could have substantial positive influences on the human consumption of salmon as a functional food.

## Methods

### Approval for animal experiments

The study was approved by the University College Dublin Animal Research Ethics Committee (approval AREC-23-01-Hulsey). All experiments were performed in accordance with relevant named guidelines and regulations and comply with the ARRIVE guidelines.

### Establishing the experimental populations

To maximize genetic diversity in the experiments, two Atlantic Salmon (*Salmo salar*) strains were used to produce juveniles for the experimental feed treatments. Alevins of both an ancestrally Norwegian strain commonly used in aquaculture and an Irish captive bred strain from the Burrishoole River in western Ireland were generated using multiple male and female parents from each strain. Alevins were held to the free-swimming fry stage and commencement of exogenous feeding. These were subsequently placed in large outside experimental enclosures supplied with a common and constantly replenishing flow-through stream-derived water at the Marine Institute Newport Research Facility in Ireland. The water was gravitationally drawn from a natural freshwater lake above the experimental enclosures and effectively replaced every few minutes. Water levels of each circular 3.6 m in diameter enclosure were maintained between a depth of approximately 30–45 cm. Experimental enclosures were maintained at ambient temperatures that ranged between 5 and 20 °C during the 6 months experimental trial. Once the fry initiated first feeding (~ 1st April 2023), they were maintained initially for 1 week on standard commercial diets and then switched to one of three experimentally formulated aquafeeds.

### Experimental feeds

The three experimental aquafeeds (50% crude protein, 21% crude lipid) varied only in the amount of the vitamin D_3_ (cholecalciferol). All feeds included a standard salmon feed base of fishmeal LT (50.0%), lysine (0.6%), methionine (1.2%), krill + squid + CPSP90 in a 4:2:4 ratio (10.0%), wheat gluten (10.0%), soybean protein concentrate (3.6%), fish oil (10.0%), canola:soybean oil in a 1:1 ratio (2.5%), soybean lecithin (1.0%), choline chloride (0.5%), betaine (0.5%), vitamin-mineral premix (2.0%) and had a content of 0.00005% cholecalciferol, vitamin C (0.1%), and calcium phosphate (0.5%). The feeds also included the cholecalciferol (vitamin D_3_) at one of three levels (1) 0 µg/kg, (2) 100 µg/kg, and (3) 1000 µg/kg. Because the vitamin-mineral premix included cholecalciferol, the control feed contained a baseline level of 729 µg/kg vitamin D_3_. Briefly, all ingredients were mixed in a 10 L mixer, ground with a hammer mill (UPZ 100, Hosokawa-Alpine, Augsburg, Germany) to 0.5 mm. The diets were extruded in a five-section twin-screw extruder (Evolum 25, Clextral, Firminy, France), fitted with 0.5 and 2 mm die holes. The pellets were dried after extrusion at 27 °C using a drying chamber (Airfrio, Almería), and cooled at room temperature. Experimental diets were formulated and elaborated by the Experimental Diet Service at the Universidad de Almeria (Spain). The three experimental feed treatments were each provided ad libitum to the respective tanks containing the developing salmon.

### Sampling

To sample the tissues for RNA-sequencing, the fish were sacrificed with an overdose of MS222 and muscles dissected during a single week 6 months after initial feeding (~ 1st October 2023). First, for ten individuals per strain for each of the three dietary treatments, the muscle commonly forming the filet from the dorsal left side of the fish was sampled and frozen at − 80 °C for subsequent vitamin D quantification. We then dissected samples from the dorsal right side of the fish corresponding to frequently consumed salmon filets (axial skeletal muscle), the adductor mandibulae (craniofacial skeletal muscle), the stomach (smooth muscle), and heart (cardiac muscle) from three individuals of each strain per treatment (3 vitamin D levels × 4 muscle tissues × 3 individuals × 2 salmon strains; n = 72). As the individuals were juveniles at the time of sampling and therefore not sexually dimorphic, sex was unable to be determined. The tissues were individually stored in RNAlater (Sigma-Aldrich) in labeled 1.5 mL tubes and frozen at − 80 °C prior to sequencing.

### Vitamin D quantification

Axial skeletal muscle filets were used to quantify vitamin D for ten samples of each strain per treatment and four samples of feed for each level. The amounts of vitamin D_3_ and 25-hydroxyvitamin D_3_ (25(OH)D_3_) were quantified using liquid chromatography, combined with triple/quadrupole mass spectrometry and electrospray ionisation (Agilent 1200 Series and Agilent 6470, Agilent Technologies, Santa Clara, CA, USA). The filet samples were stored initially at − 20 °C for 1 week and then transferred to a − 80 °C freezer until quantification at a maximum of 10 months following tissue sampling. The limit of quantification (LOQ) was 1 ng/g for vitamin D_3_ and 25-hydroxyvitamin D_3_ (25(OH)D_3_). All filet samples were analysed by single determination, and precision was based on an in-house control sample of salmon run 14 times during the analyses for the whole study that showed a precision of 5.4% for vitamin D_3_. The same protocol was used to quantify vitamin D_3_ levels in one sample each of the experimental feeds. A two-way ANOVA performed in R v4.4.1^56^ was used to test for differences in vitamin D_3_ accumulation in the filet among the three vitamin D treatments and the two strains. Tukey post-hoc tests were used to evaluate the pairwise-comparisons between the group means.

### Body condition

Because vitamin D could influence fish either negatively or positively, the size-adjusted body condition of fish were measured. Towards the end of the experiment (5 September 2023), a few weeks before fish were sacrificed for gene expression, a sample of 20 fish per strain were removed from each enclosure. The sampled fish were digitally photographed on 1mm graph paper. The linear fork-length of individual fish were then measured using ImageJ^57^. The mass of these same individuals were also determined using a digital scale. Subsequently, these two measurements were combined to estimate Fulton’s condition factor for each fish where fish mass was divided by the fork length raised to the third power^58^. This metric provides a common and relatively size-independent estimate of the health of fishes. A two-way ANOVA was used to test for differences in mean Fulton’s condition score among the three vitamin D treatments and the two strains, and Tukey post-hoc tests were used to evaluate pairwise-comparisons between group means.

### RNAseq library preparation and sequencing

For RNA extraction, mRNA isolation, RNA-seq library preparation, and sequencing, 72 samples were shipped on dry ice to Novogene’s Cambridge Sequencing Centre (Cambridge, UK). To prepare sequencing libraries, messenger RNA was purified from total RNA using poly-T oligo-attached magnetic beads. After fragmentation, the first strand cDNA was synthesized using random hexamer primers, followed by the second strand cDNA synthesis^59^. Following end repair, A-tailing, adapter ligation, size selection, amplification, and purification, libraries were checked with Qubit and real-time PCR for size distribution. Quantified libraries were then pooled and sequenced on the Illumina NovaSeq X Plus (PE 150) platform to generate approximately 12G raw data per sample.

### Gene expression quantification

Raw sequence reads in fastq format were initially processed through Novogene in-house perl scripts. Clean reads were obtained by removing those reads containing adapters, exhibiting poly-Ns, or displaying low-quality. Reference genome and gene model annotation files for *Salmo salar (*Ssal_v3.1) were downloaded from the Ensembl database and the index was built using Hisat2 v2.0.5^60^ from the paired-end clean reads aligned to this reference genome. FeatureCounts v1.5.0-p3^61^ was used to count the read numbers mapped to each gene. Then each gene’s FPKM, the expected number of Fragments Per Kilobase of transcript sequence per Millions base pairs sequenced, was calculated based on the gene length and reads counts mapped to this gene.

### Differential expression

Differential expression^62^ analyses of two conditions (high versus control and moderate versus control) with six biological replicates per condition was performed using the DESeq2 R package v 1.20.0^63^. Pre-filtering was performed to keep only rows that had a count of at least 10 for a minimum of three samples. The DESeq2 design included vitamin D treatment as the condition while controlling for strain as a batch effect. The resulting *p*-values were adjusted using the Benjamini and Hochberg’s approach to controlling the false discovery rate^64^. Genes with an adjusted *p*-value less than or equal to 0.05 found by DESeq2 were assigned as differentially expressed. To improve effect size estimation, log_2_FC changes were shrunk using the lfcShrink() function with the apeglm method^65^.

### GO and KEGG enrichment

Gene Ontology (GO) enrichment analyses^66^ of the large number of differentially expressed genes in the heart was implemented by the clusterProfiler R package v4.12.3^67,68^. GO terms with adjusted *p*-value less than or equal to 0.05 were considered significantly enriched in differentially expressed genes. The KEGG database (http://www.genome.jp/kegg/) was also queried to examine high-level functional differences of gene expression profiles^69–71^. We used the clusterProfiler R package to test the statistical enrichment of differential gene expression in KEGG pathways^69^.

### Salmon and mammal gene expression homology

Because of the salmonid specific genome duplication, many of the genes in the *S. salar* genome have not been annotated on Ensembl. We therefore commonly refer to genes via their abbreviated Ensembl IDs (e.g. ENSSSAG00000116104 abbreviated to EX116104). However, to provide our own annotations of loci, we first searched the Ensembl IDs on NCBI gene. If that failed to provide a description, we subjected the largest exon for each gene in Ensembl to a NCBI BLAST nucleotide database search. If a BLAST search returned a gene that scored over 80% identity and query cover, we added that gene annotation to our Ensembl gene ID list.

Finally, we determined which differentially expressed salmon genes showed records of homologous loci being differentially expressed in mammalian muscle in response to vitamin D. For this annotation, we implemented the research utility of the NCBI E-utilities API^72^. We shortened the gene names to remove any identifiers of teleost and salmonid specific genome duplicates (e.g., adcy6b became adcy6). For each gene that had a gene name (542 genes), we then searched “‘[gene name]’ AND expression AND muscle AND ‘vitamin D’” to determine which of our differentially expressed genes are influenced by vitamin D titers in other vertebrates. All results were then manually checked for accuracy and relevance.

## Supplementary Information

Below is the link to the electronic supplementary material.

Supplementary Material 1

## Data Availability

The RNA sequencing data has been submitted to the NCBI short-read archive (PRJNA1160017). All other datasets generated during and/or analyzed during the current study are available from the corresponding author on reasonable request.
